# Supplementary material for: Acute deletion of TET enzymes results in aneuploidy in mouse embryonic stem cells through decreased expression of Khdc3
Source: Nat Commun. 2022 Oct 20;13:6230. doi: 10.1038/s41467-022-33742-7 (PMC9584922; doi:10.1038/s41467-022-33742-7)
Supplement: Supplementary file 1 — Supplementary Information [file 41467_2022_33742_MOESM1_ESM.pdf]

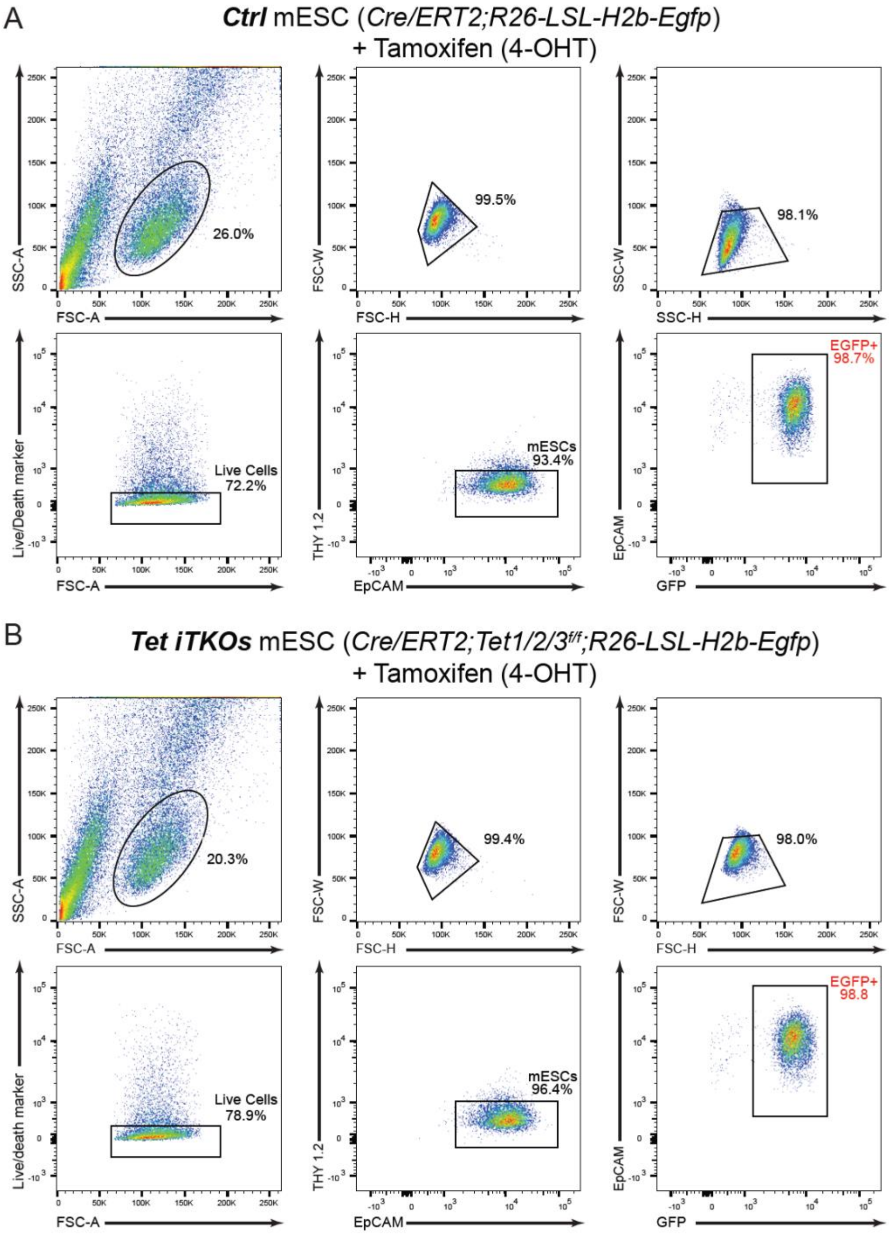

Supplementary Figure 1: Fluorescence activated cell sorting (FACS) analysis of *Ctrl* and *Tet iTKO* mESC populations. Live single cells gated using Live/Dead staining show low levels of the MEF marker CD90.2 (THY1.2) and high levels of the ESC marker CD326 (EpCAM). Activation of the Cre/ERT2 system after Tamoxifen treatment was detected by the expression of the H2b-EGFP reporter (GFP) contained in the *Rosa26* locus. Both *Ctrl* (A) and *Tet iTKO* (B) mESC populations were >98% EGFP$^+$ after treatment with 4-OHT. The EGFP+ cells were sorted and used for further analysis of *Ctrl* and *Tet iTKO* mESC samples.

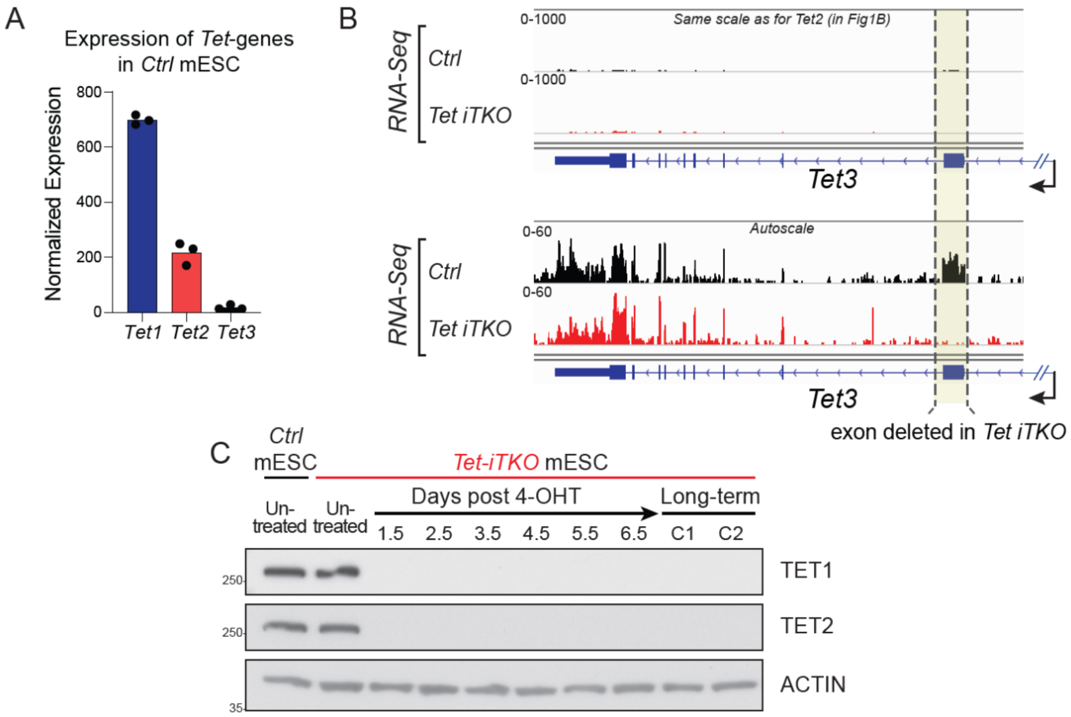

Supplementary Figure 2: TET protein levels are rapidly and efficiently decreased after tamoxifen treatment of *Tet iTKO* mESC. (**A**) Normalized expression of *Tet1*, *Tet2* and *Tet3* genes in *Ctrl* mESC, showing insignificant expression of *Tet3*. (**B**) *Top*, *Tet3* is poorly expressed in both *Ctrl* and *Tet iTKO* mESC after tamoxifen treatment (plotted on the same scale as for *Tet2* in Fig. 1B); *bottom*, *Tet3* expression plotted on an expanded scale (1-60 instead of 1-1000 in Fig. 1B) to show efficient deletion of the targeted region of *Tet3* (highlighted in yellow) in *Tet iTKO* compared to *Ctrl* mESC. (**C**) Western blot analysis showing rapid loss of TET1 and TET2 proteins after tamoxifen treatment. *Ctrl* mESC as well as untreated *Tet iTKO* mESC were included as controls. *Tet iTKO* mESC were collected at the indicated times (1.5 to 6.5 days after withdrawal of 4-OHT). Two clones of long-term *Tet iTKO* mESC were also included (indicated as C1 and C2). ACTIN was used as loading control.

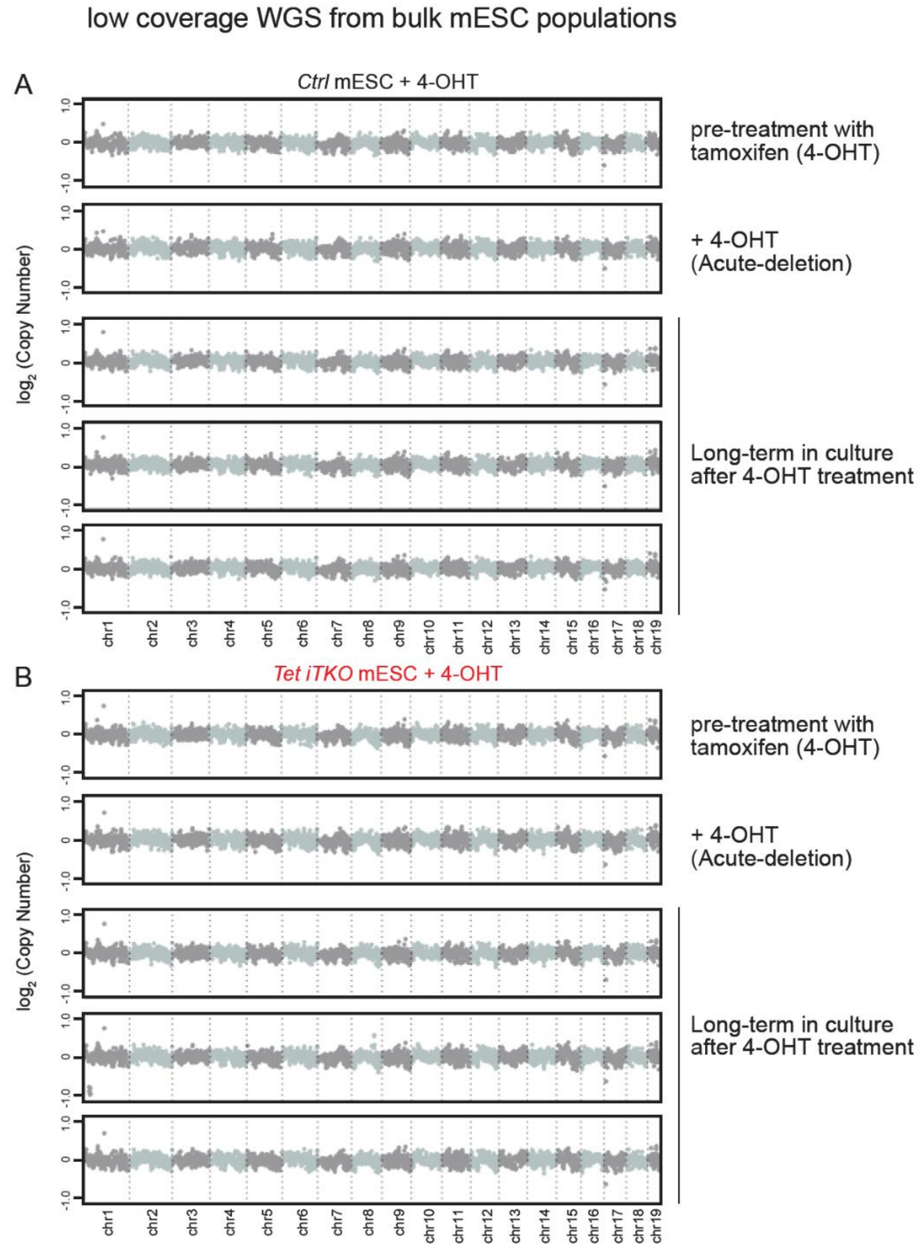

Supplementary Figure 3: Apparent euploid karyotype of bulk *Tet iTKO* mESC populations. Data from low coverage Whole-Genome Sequencing (WGS) of **A**, bulk *Ctrl* and **B**, bulk acutely-deleted *Tet iTKO* 6.5 days after 4-OHT treatment. Each dot represents a 500 kb window. As shown in this study, single-cell analysis (by metaphase spreads) is needed to detect the stochastic appearance of aneuploidies.

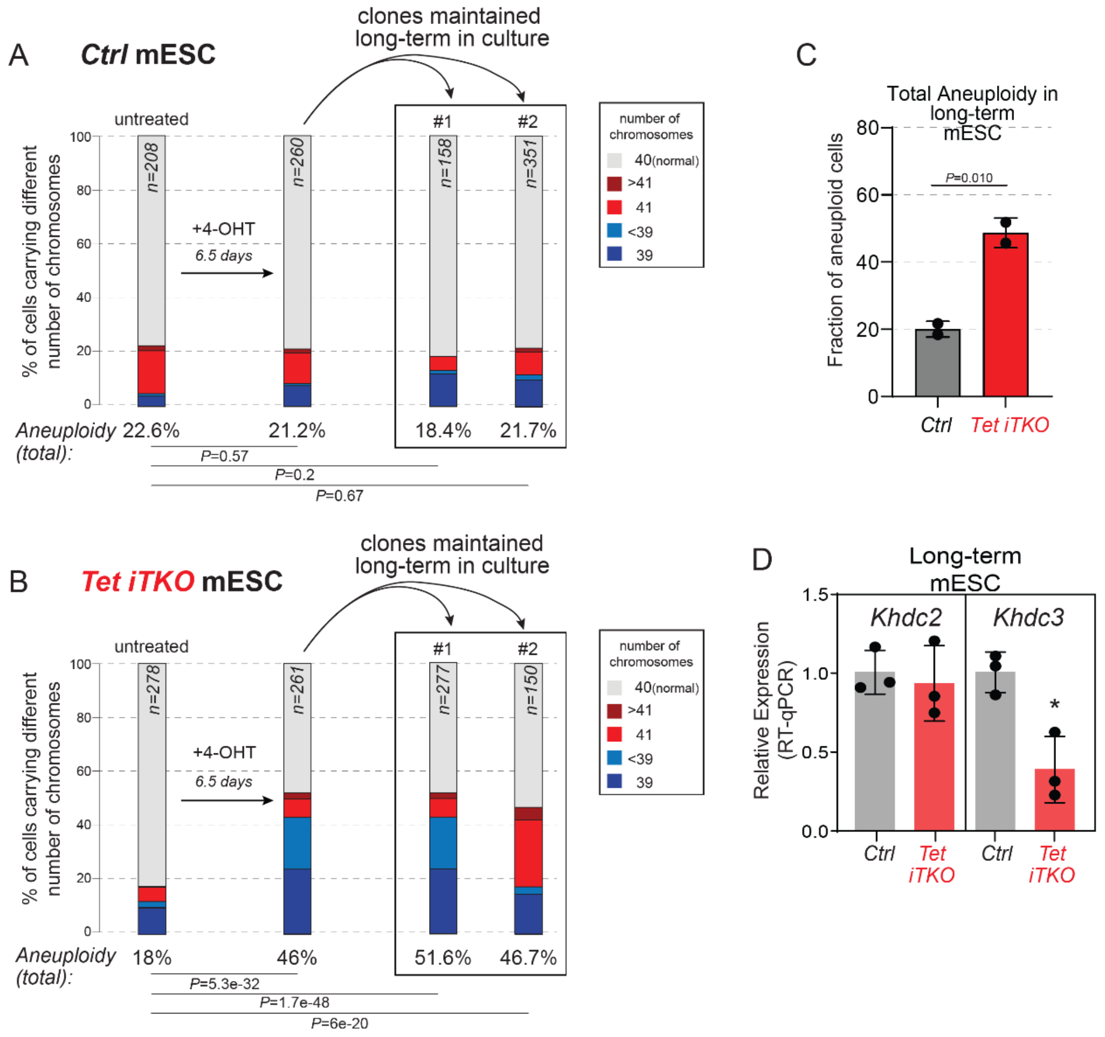

Supplementary Figure 4. TET-deficient mESC maintained for long periods in culture still display aneuploidy. *Ctrl* (**A**) and *Tet Tfl* (**B**) mESC lines were treated with 4-OHT for 2.5 days, then sorted using fluorescence-activated cell sorting (FACS) for GFP^+ cells on day 6.5 to derive clonal mESC populations. The clones derived after sorting were maintained for an additional 50 days in culture (>80 cell divisions equivalent to >15 Passages) in the absence of 4-OHT, and aneuploidy was assessed by metaphase spreads in bulk cultures of the parental unstimulated and 4-OHT-treated *Ctrl* and *Tet iTKO* mESC (*left*) as well as in two clones derived from each *Ctrl* and *Tet iTKO* culture (*right*). The increase in aneuploidy seen after acute *Tet* gene deletion was maintained in long-term culture. Chi-square test was performed, and the calculated p-values are included for each indicated comparison. (**C**) Bar graph +/- standard error summarizing the data from cells treated with 4-OHT in **A** and **B** that were maintained in long-term culture (n=2). Statistical differences between two combined *Ctrl* mESC populations (*Ctrl* mESC #1 and #2) and two *Tet iTKO* mESC populations (*Tet iTKO* mESC #1 and #2) were evaluated using Chi-square test. (**D**) Relative mRNA expression of *Khdc2* and *Khdc3* in long-term cultured *Ctrl* (gray) and *Tet iTKO* (red) mESC. Note that only *Khdc3* remains repressed after long-term culture of *Tet iTKO* mESC, whereas expression of *Khdc2* returns to normal levels (n=3). Bar graphs represent the mean +/- standard deviation. Statistical analysis was performed using unpaired t-test (*<0.05).

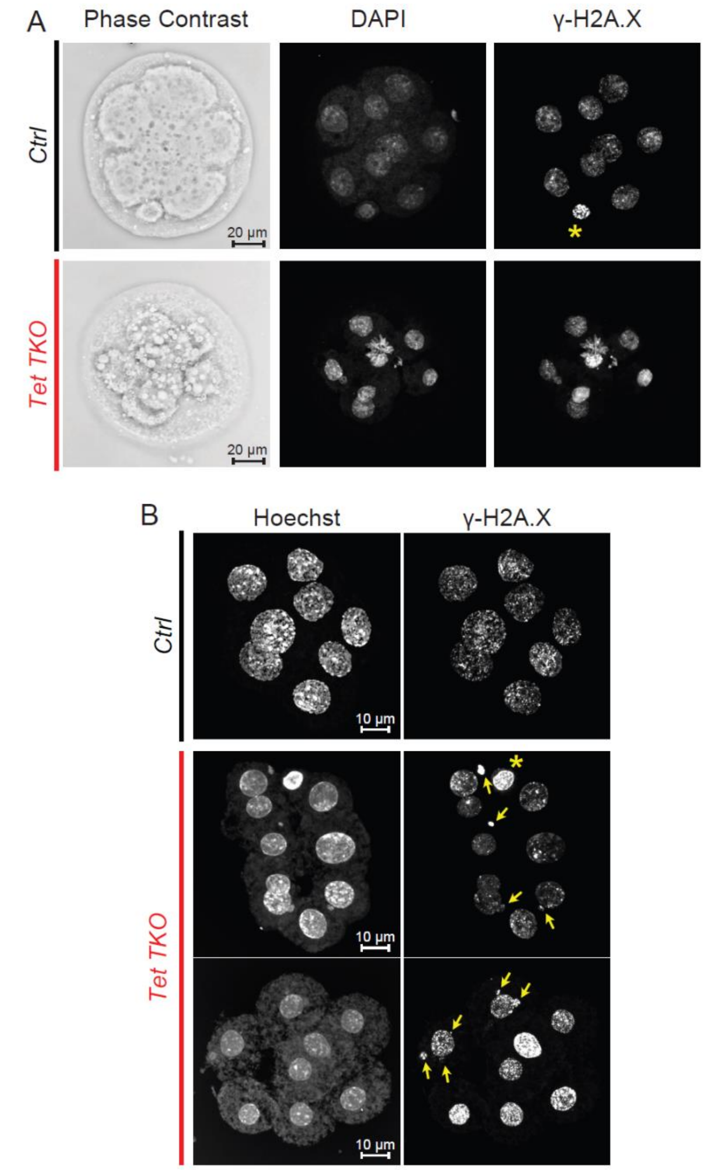

Supplementary Figure 5: Immunofluorescent and phase contrast images of *Ctrl* and *Tet TKO* embryos. (**A**) Phase contrast images (*left*) from the same *Ctrl* and *Tet TKO* embryos shown in Fig. 2F were supplemented to the immunofluorescent staining of DAPI (middle) and γ-H2A.X (*right*) staining in grayscale. (**B**) An independent group of embryos was analyzed by confocal microscopy (Airyscan) using Hoechst (*left*) and γ-H2A.X (*right*) staining. Yellow arrows and asterisks highlight micronuclei and polar bodies respectively.

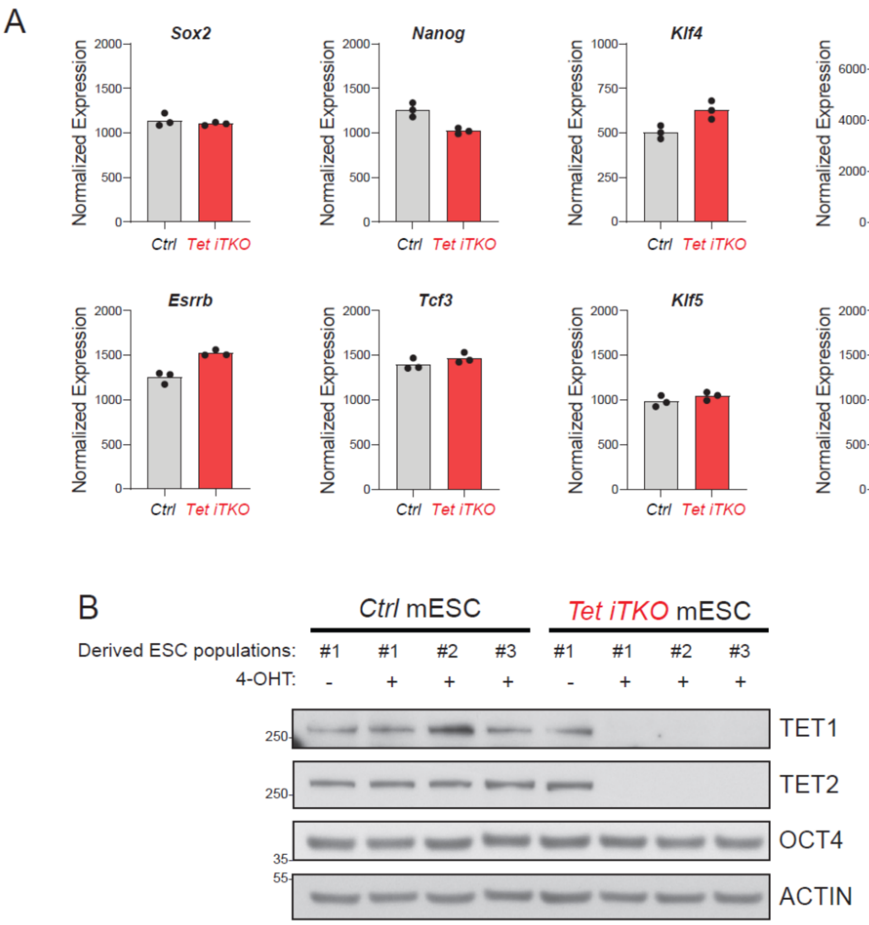

Supplementary Figure 6: Acute *Tet*-deletion in mESC does not significantly affect the expression of genes related to pluripotency or stem cell maintenance. (**A**) Normalized expression of genes related to pluripotency (*top*) and stem cell maintenance (*bottom*) from mRNA-seq (*this study*). (B) Western blot analyses of three different mESC populations derived from three different embryos show consistent effective depletion of TET1 and TET2 proteins after tamoxifen treatment as well as unaltered levels of the pluripotency marker OCT4 in all populations. ACTIN was used as the loading control.

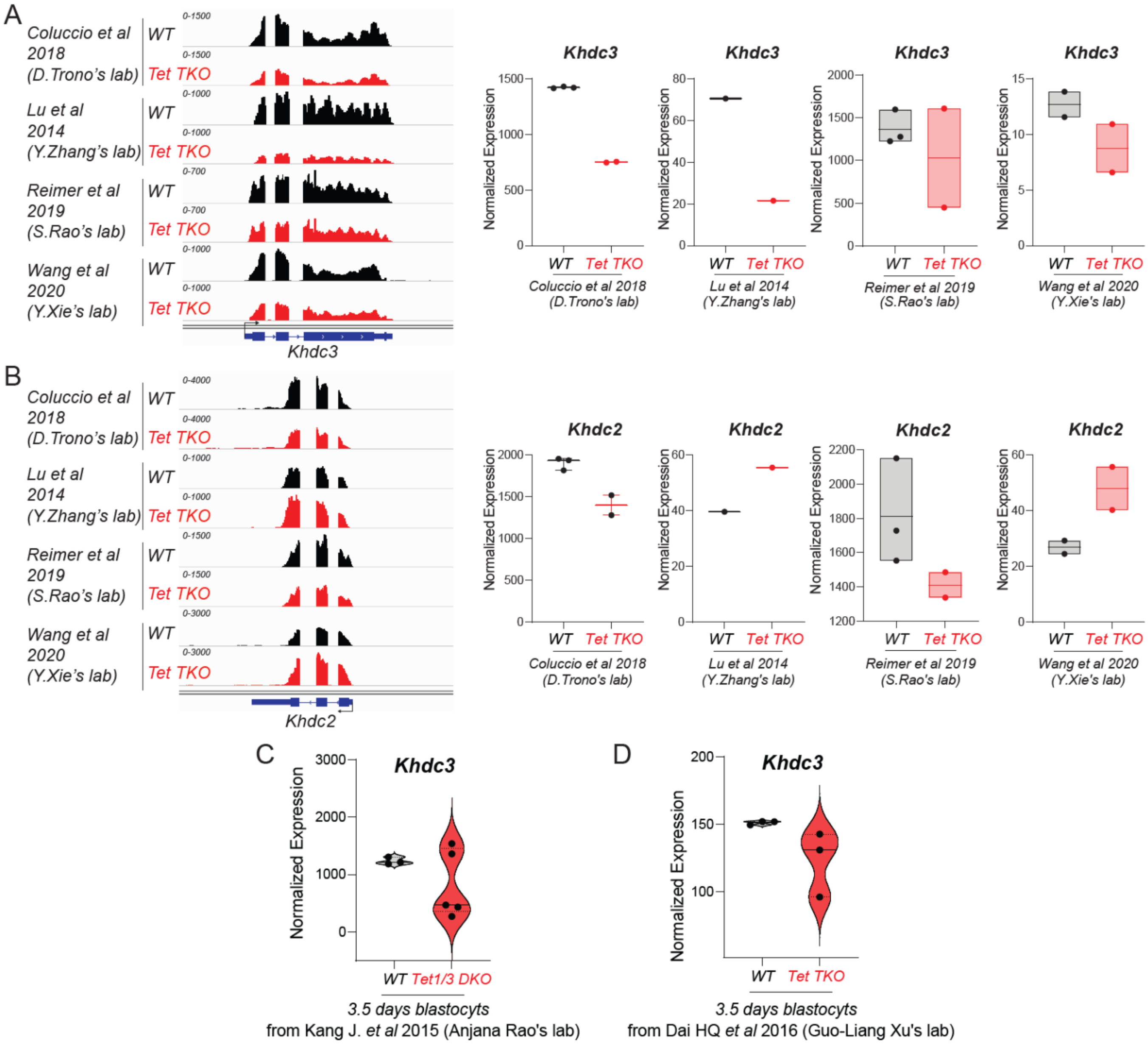

Supplementary Figure 7: TET proteins are required to sustain the normal expression of *Khdc3* in mESC and early embryos. A, **B**, Expression of *Khdc3* (**A**) and *Khdc2* (**B**) in constitutive *Tet* triple KO (*Tet TKO*) mESC compared to *WT*. *Left*, Genome browser views of the *Khdc3* and *Khdc2* genes taken from four published studies [**51, 59-61**]. *Right*, Normalized expression of *Khdc3* and *Khdc2* in each study compared to their corresponding controls (*WT*). The number of samples from each study were (from *left* to *right*) 5, 2, 5 and 4. Bar graphs represent the mean +/- standard deviation. (**C**) Transcriptomes from five *Tet1/3* DKO blastocysts (3.5 days) were analyzed and compared to three wildtype (*WT*) blastocysts [**35**]. 3 of 5 *Tet1/3* DKO blastocytes failed to maintain normal levels of *Khdc3*. (**D**) Three blastocysts lacking all TET enzymes (*Tet TKO*) [**49**] were compared to the same number of control (*WT*) blastocysts. All *Tet TKO* blastocysts have reduced levels of *Khdc3* compared to their controls.

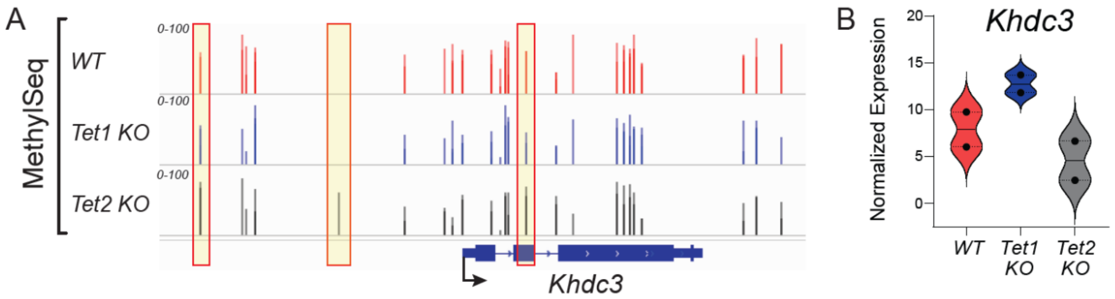

Supplementary Figure 8: TET2 maintains DNA demethylation at the *Khdc3* promoter. (**A**) Genome browser view of the *Khdc3*-loci in mESC. The DNA methylation states of *WT* (red), *Tet1 KO* (blue) or *Tet2* KO (black) mESC were determined by WGBS [**102**]. (**B**) The mRNA expression level of *Khdc3* in the same mESC lines was determined by RNA-seq. Note that *Khdc3* is down-regulated in *Tet2 KO* but not *Tet1 KO* mESC compared to *WT*.

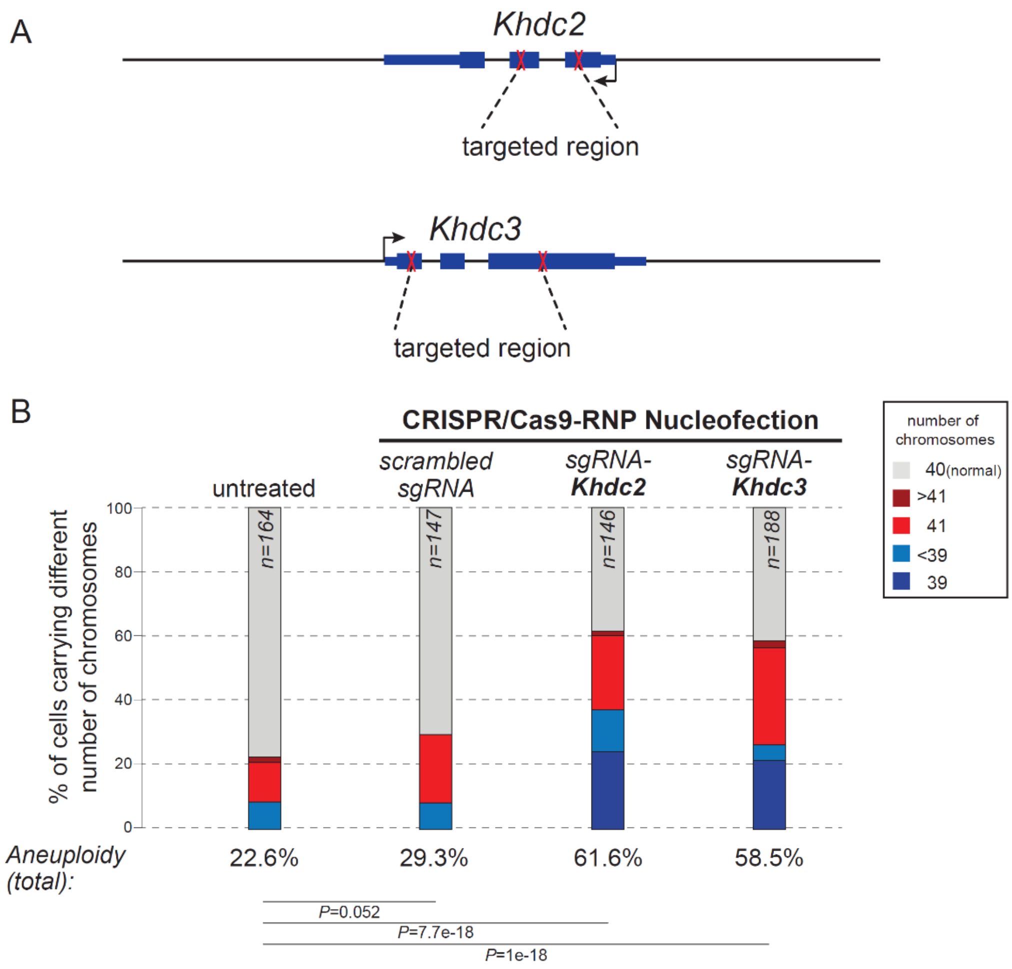

Supplementary Figure 9: Disruption of the *Khdc2* or *Khdc3* genes increase the emergence of aneuploidies in mESC. A, Schematic representation CRISPR/Cas9-mediated editing of targeted regions depicted in red within *Khdc2* and *Khdc3* exons using two single guide RNAs (sgRNAs) per gene. CRISPR/Cas9-RNP delivery by nucleofection into mESC led to highly efficient *Khdc2* and *Khdc3* gene disruption. Editing of targeted regions in the *Khdc2* and *Khdc3* genes was confirmed by Sanger sequencing. **B**, Metaphase Spreads analyses of *Khdc2*-KO (sgRNA-*Khdc2*) and *Khdc3*-KO (sgRNA-*Khdc3*) mESC were performed as in Figures 2 and 5. For controls, non-nucleofected mESC (untreated) as well as nucleofected mESC with an Universal Non-Targeting Control sgRNA (scrambled sgRNA) were also included. The number of counted cells is indicated in each case. Statistical differences were calculated relative to untreated *Ctrl* mESC using the Chi-square test (p-values are indicated for each comparison).

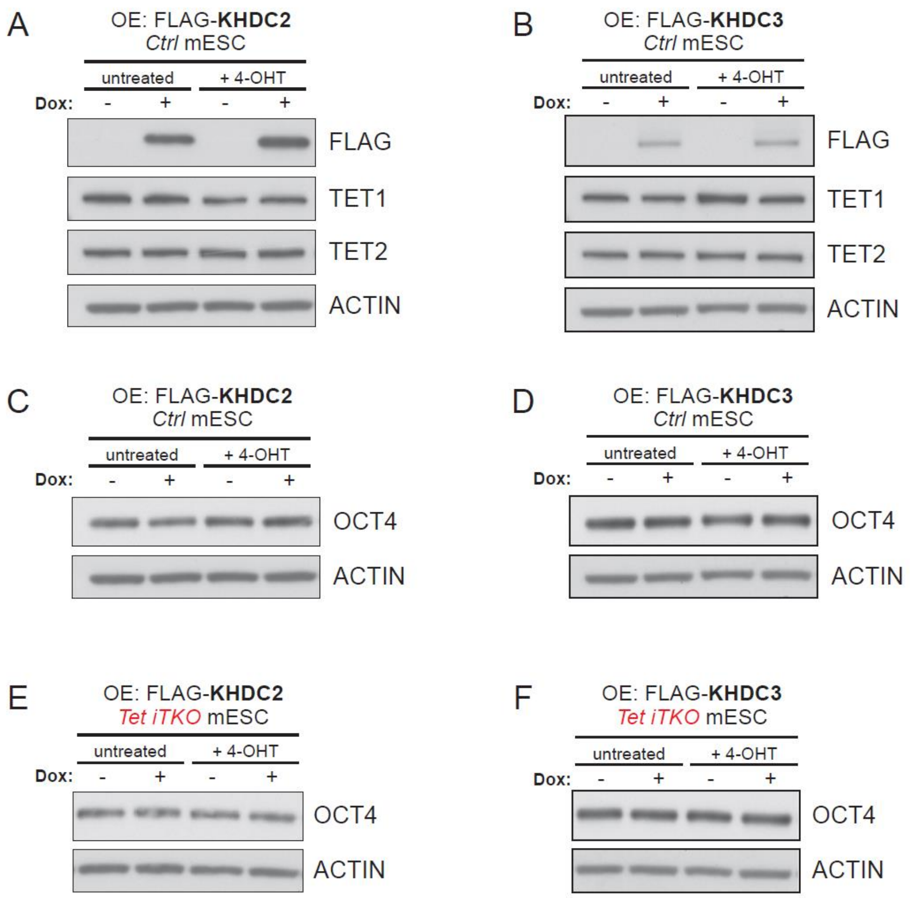

Supplementary Figure 10: Expression of epitope-tagged Flag-KHDC2 and Flag-KHDC3 proteins in *Ctrl* and *Tet iTKO* mESC does not affect TET1, TET2, nor OCT4 expression. Western blots of *Ctrl* mESC carrying the doxycycline (Dox) inducible system presented in Figure 5 to overexpress (**A**) Flag-KHDC2 or (**B**) Flag-KHDC3. Levels of TET1 and TET2 remain unaltered in the *Ctrl* mESC after tamoxifen-treatment, as expected. Western blot analyses for the pluripotency marker OCT4 in *Ctrl* (**C-D**) or *Tet iTKO* (**E-F**) mESC carrying the inducible systems to overexpress KHDC2 or KHDC3. OCT4 protein levels were unchanged. In all panels, ACTIN was used as loading control.

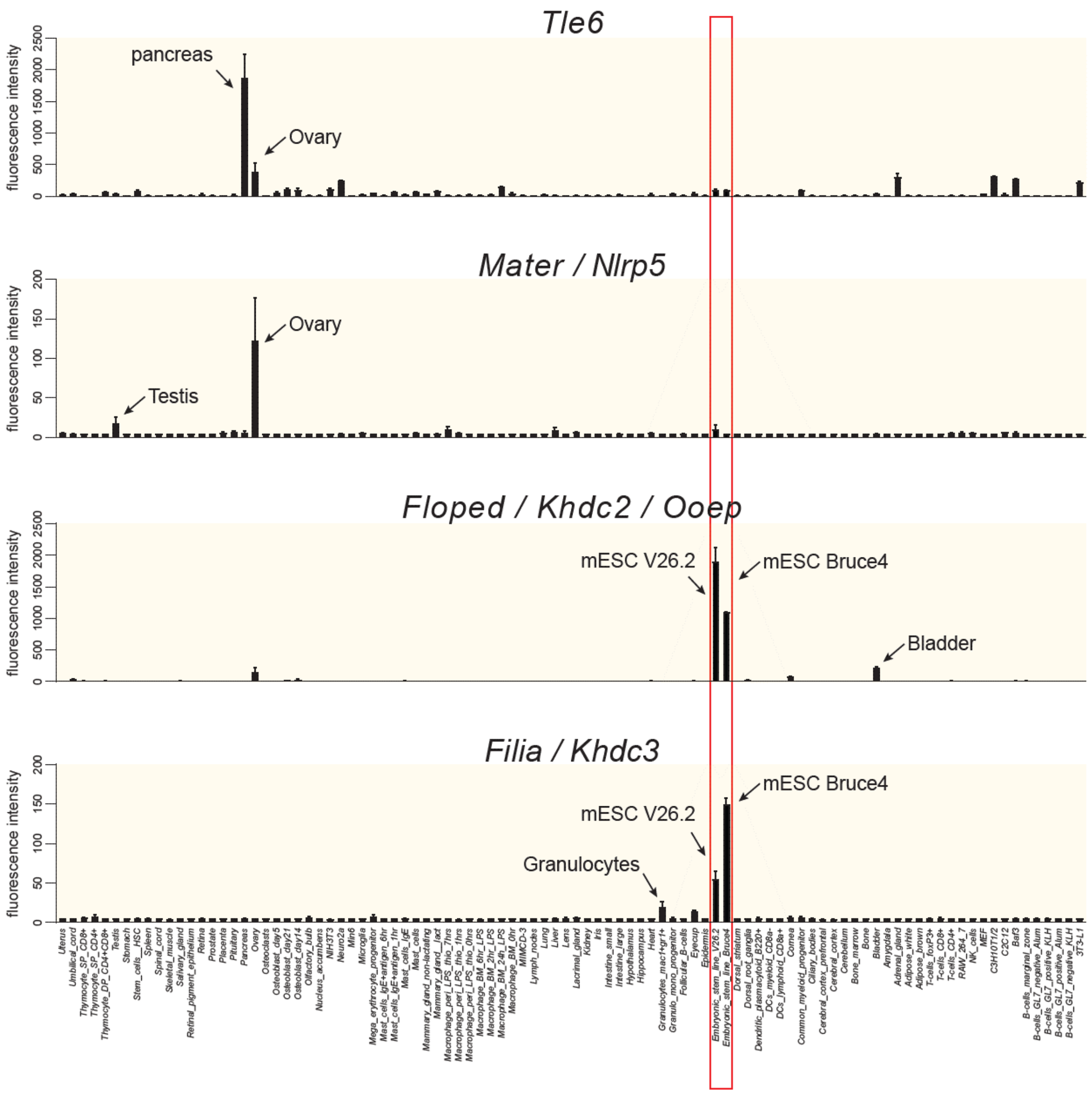

Supplementary Figure 11: Gene expression profiles of *Khdc3* and the core subunits of the Subcortical Maternal Complex (SCMC). Gene expression levels of the indicated genes were extracted from GeneAtlas MOE430, which profiles 191 samples from 96 different tissues, cell types and experimental conditions. Samples from mouse Embryonic Stem Cells (v26 and Bruce4) are highlighted in red. Data were normalized using the Background Adjustment Using Sequence Information method (gcrma).

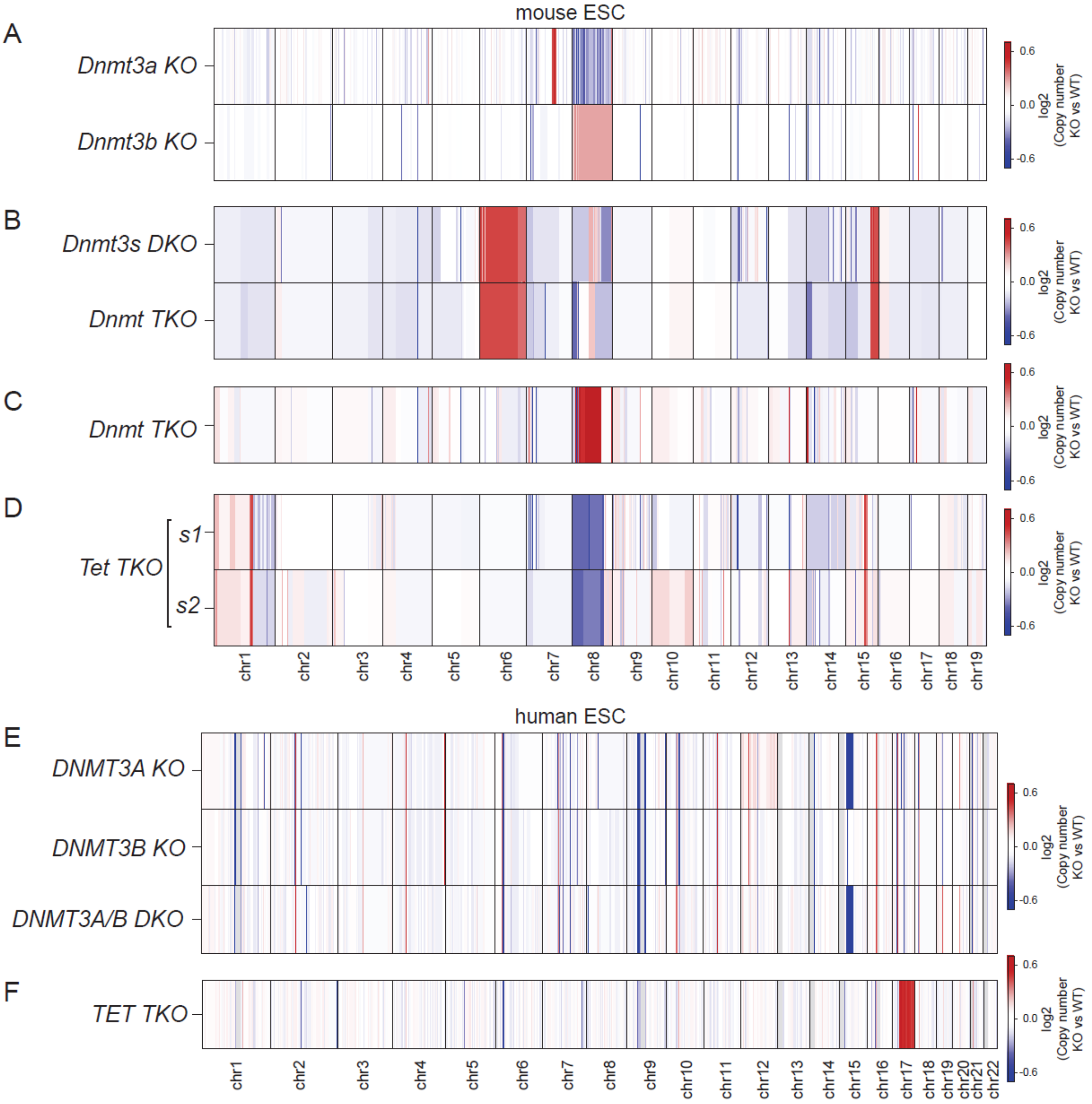

Supplementary Figure 12: Disruption of *Tet* and *Dnmt* genes results in aneuploidies in mESC. Whole Genome Bisulfite Sequencing (WGBS) data from different mESC lines carrying single (**A**) [**71**], double or triple (**B**) [**72**]) *Dnmt* gene knockouts were used to analyze gains (red) and losses (blue) of chromosomal regions compared to their parental *wild-type* mESC cell lines. (**C**) The *Dnmt TKO* mESC line used in this study (*shown in Fig1*) also showed aneuploidies. (**D**) Two replicates from constitutive *Tet TKO* models [**61**] were also analyzed using high coverage WGBS datasets and compared against their parental lines. (**E**) human ESC samples lacking *DNMT3A, DNMT3B* or both genes [**103**] were analyzed using similar strategies. Single *DNMT3A* (but not *DNMT3B*) deletion generated a copy number change in chromosome 15 that was also detected in *DNMT3A/B DKO* hESCs. (**F**) High coverage WGBS samples from constitutive human *TET TKO* ESCs [**67**] revealed the persistent appearance of aneuploidies also in human TET-deficient cells.
