# Supplementary material for: Acute deletion of TET enzymes results in aneuploidy in mouse embryonic stem cells through decreased expression of Khdc3
Source: Nat Commun. 2022 Oct 20;13:6230. doi: 10.1038/s41467-022-33742-7 (PMC9584922; doi:10.1038/s41467-022-33742-7)
Supplement: Supplementary file 3 — Reporting Summary [file 41467_2022_33742_MOESM3_ESM.pdf]

nature portfolio

Corresponding author(s): Anjana Rao
Last updated by author(s): Aug 23, 2022

Reporting Summary

Nature Portfolio wishes to improve the reproducibility of the work that we publish. This form provides structure for consistency and transparency in reporting. For further information on Nature Portfolio policies, see our Editorial Policies and the Editorial Policy Checklist.

Statistics

For all statistical analyses, confirm that the following items are present in the figure legend, table legend, main text, or Methods section.

n/a	Confirmed	
☐	☒	The exact sample size (*n*) for each experimental group/condition, given as a discrete number and unit of measurement
☐	☒	A statement on whether measurements were taken from distinct samples or whether the same sample was measured repeatedly
☐	☒	The statistical test(s) used AND whether they are one- or two-sided<br>*Only common tests should be described solely by name; describe more complex techniques in the Methods section.*
☐	☒	A description of all covariates tested
☒	☐	A description of any assumptions or corrections, such as tests of normality and adjustment for multiple comparisons
☐	☒	A full description of the statistical parameters including central tendency (e.g. means) or other basic estimates (e.g. regression coefficient) AND variation (e.g. standard deviation) or associated estimates of uncertainty (e.g. confidence intervals)
☐	☒	For null hypothesis testing, the test statistic (e.g. *F*, *t*, *r*) with confidence intervals, effect sizes, degrees of freedom and *P* value noted<br>*Give P values as exact values whenever suitable.*
☒	☐	For Bayesian analysis, information on the choice of priors and Markov chain Monte Carlo settings
☒	☐	For hierarchical and complex designs, identification of the appropriate level for tests and full reporting of outcomes
☒	☐	Estimates of effect sizes (e.g. Cohen's *d*, Pearson's *r*), indicating how they were calculated

Our web collection on statistics for biologists contains articles on many of the points above.

Software and code

Policy information about availability of computer code

Data collection	For Flow Cytometry, the data was collected using BD FACSDiva software (version 8.0.2) on BD LSR II, LSR Fortessa. For Fluorescence Live-Cell Imaging, we used the Confocal Quantitative Image Cytometer CQ1 benchtop high-content analysis system (Yokogawa). For Immunocytochemistry, we used Airyscan microscope with Zeiss ZEN software.
Data analysis	Flow Cytometry: All data was analyzed using Flow Jo software (TreeStar, version 9.7.6 and 10.3.0).<br>Graphs and statistics: GraphPad Prism Version 9 was used to plot graphs, mean with standard deviation was shown on each graph. The statistical significance was performed using two-tailed unpaired student's t test.<br>For RNA-seq analysis, reads were aligned using STAR (Dobin et al 2013) with the parameters --outFilterMultimapNmax 1, --outSAMtype BAM SortedByCoordinate –sjdbOverhang 100. HT-Seq (Anders, S. et al 2015) was used to quantify the gene expression levels using the options htseq-count -s yes, -r pos, -a 10. Normalization and differential expression analyses were performed using DESEq2 (Love M. et al 2014), with the parameters fitType parametric, alpha 0.05 and using the Benjamini & Hochberg method. For visualization of the data, we generated tracks using Deeptools (Ramírez, F. et al 2014), with the option bamCoverage. All related plots were made using R-Studio (Team, R. C. 2013) and Integrative Genome Viewer (IGV) (Thorvaldsdóttir, H. et al 2013).<br>For ChIP-Seq analyses of published datasets (Gene Expression Omnibus (GEO)), we used Bowtie (Langmead et al 2009, 19261174) and Deeptools with the option bamCoverage. For analyses of DNA methylation, we analyzed Whole Genome Bisulfate sequencing (WGBS) samples from the GEO database using Bismark (Krueger, F. et al 2011) and bedGraphToBigWig for tracks generation.

For manuscripts utilizing custom algorithms or software that are central to the research but not yet described in published literature, software must be made available to editors and reviewers. We strongly encourage code deposition in a community repository (e.g. GitHub). See the Nature Portfolio guidelines for submitting code & software for further information.

Data

Policy information about availability of data

All manuscripts must include a data availability statement. This statement should provide the following information, where applicable:

- Accession codes, unique identifiers, or web links for publicly available datasets
- A description of any restrictions on data availability
- For clinical datasets or third party data, please ensure that the statement adheres to our policy

Our RNA-seq data is available with the following accession number: GSE191045

Human research participants

Policy information about studies involving human research participants and Sex and Gender in Research.

Reporting on sex and gender	n/a
Population characteristics	n/a
Recruitment	n/a
Ethics oversight	n/a

Note that full information on the approval of the study protocol must also be provided in the manuscript.

Field-specific reporting

Please select the one below that is the best fit for your research. If you are not sure, read the appropriate sections before making your selection.

☒ Life sciences ☐ Behavioural & social sciences ☐ Ecological, evolutionary & environmental sciences

For a reference copy of the document with all sections, see nature.com/documents/nr-reporting-summary-flat.pdf

Life sciences study design

All studies must disclose on these points even when the disclosure is negative.

Sample size	Sample size was determined by previous studies or pilot studies. No statistical test was performed to predetermine sample size. All experiments were performed using 3 mESC populations (different biological replicates, from 3 different embryos) for each genotype. For metaphase spreads, more than 7000 cells were counted in total, with an average of 255 cells per experiment.<br>All experiments were repeated at least 3 times unless is indicated in the figure legends.
Data exclusions	No data was excluded from the analysis
Replication	All data were successfully reproduced with replicates for each experiment.
Randomization	Experimental groups were chosen based on genotypes, and treatments (tamoxifen treatments). All animals selected for processing, imaging, and analysis were randomly chosen with no bias. Sex and age matched animals were used for experimental and control groups. All derived mESC (from 3 different embryos for each genotype) were used without bias.
Blinding	The experiments were not blinded, with the exception of metaphase spread analyses, where the observers were blinded about the group designations.

Reporting for specific materials, systems and methods

We require information from authors about some types of materials, experimental systems and methods used in many studies. Here, indicate whether each material, system or method listed is relevant to your study. If you are not sure if a list item applies to your research, read the appropriate section before selecting a response.

Materials & experimental systems

n/a	Involved in the study
☐	☒ Antibodies
☐	☒ Eukaryotic cell lines
☒	☐ Palaeontology and archaeology
☐	☒ Animals and other organisms
☒	☐ Clinical data
☒	☐ Dual use research of concern

Methods

n/a	Involved in the study
☒	☐ ChIP-seq
☐	☒ Flow cytometry
☒	☐ MRI-based neuroimaging

Antibodies

Antibodies used	Brilliant Violet 510™ anti-mouse CD90.2 (Thy-1.2) Antibody (BioLegend, 140319; dilution 1:250); Brilliant Violet 605™ anti-mouse CD326 (Ep-CAM) Antibody (BioLegend, 118227; dilution 1:250); anti-Tet1 (Millipore-Sigma 09-872; dilution 1:4000); anti-Tet2 (Abcam, ab124297; dilution 1:4000); β-Actin (13E5) Rabbit mAb (HRP Conjugate) (Cell Signaling, 5125S; dilution 1:10000); anti-5hmC (Active Motif, 39769; dilution 1:1000). anti-Flag M2 (Millipore-Sigma, F3165; dilution 3000).
Validation	For Tet-antibodies we validated them using our own Knock-out system.<br>All other used used on this study are commercially availables and validated from their companies. Validation was based on data sheets provided by manufacturers and was also confirmed in the literature. Any information about the validation performed by the manufacturer can be downloaded in the manufacturers' websites.

Eukaryotic cell lines

Policy information about cell lines and Sex and Gender in Research

Cell line source(s)	mESC were directly derived derived from mice; Lenti-XTM 293T cell line (Takara, 632180)
Authentication	mESC were characterized by morphology, genotyping (PCR), colony formation, expression of pluripotency markers (RT-PCR and RNA-seq), self-renewal and differentiation potential.
Mycoplasma contamination	All cell lines tested negative for mycoplasma contamination.
Commonly misidentified lines (See ICLAC register)	No commonly misidentified lines were used.

Animals and other research organisms

Policy information about studies involving animals; ARRIVE guidelines recommended for reporting animal research, and Sex and Gender in Research

Laboratory animals	CreERT2; Rosa26-H2B-EgfpLSL mice, where LSL denotes the loxP-STOP-loxP cassette that permits expression of the floxed exons or the reporter gene only after excision by the Cre recombinase), we crossed B6.Cg-Tg(UBC-cre/ERT2)1Ejb/J mice (Jackson Laboratory, #008085), harboring the CreERT2 fusion gene under control of the human ubiquitin C (UBC) promoter, with Gt(ROSA)26Sor<tm1Ytchn>/J (Jackson Laboratory, #021847) mice containing a CRE-recombinase inducible dual reporter construct in which H2b-Egfp and GPI-mCherry transgenes were inserted into the Gt(ROSA)26Sor locus downstream of a loxP-flanked STOP fragment. The Tet-triple floxed (Tet Tfl) mouse strain (CreERT2; Tet1fl/fl; Tet2fl/fl; Tet3fl/fl; Gt(ROSA)26Sor<tm1Ytchn>/Jwt/ki) harbours CreERT2 and H2b-Egfp; GPI-mCherry transgenes, as well as the floxed alleles for all three Tet genes.<br>All mice were on the B6 background and maintained in a specific pathogen-free animal facility in the La Jolla Institute for Immunology. All animal procedures were reviewed and approved by the Institutional Animal Care and Use Committee of the La Jolla Institute for Immunology and were conducted in accordance with institutional guidelines.
Wild animals	no wild animals were used in the study.
Reporting on sex	Sex of the animals was not considered in the experimental design.
Field-collected samples	no field-collected samples were used in the study.
Ethics oversight	Mice were housed in a pathogen-free animal facility at the La Jolla Institute for Immunology and were used according to protocols approved by the Institutional Animal Care and Use Committee (IACUC).

Note that full information on the approval of the study protocol must also be provided in the manuscript.

Flow Cytometry

Plots

Confirm that:

☒ The axis labels state the marker and fluorochrome used (e.g. CD4-FITC).

☒ The axis scales are clearly visible. Include numbers along axes only for bottom left plot of group (a 'group' is an analysis of identical markers).

☒ All plots are contour plots with outliers or pseudocolor plots.

☒ A numerical value for number of cells or percentage (with statistics) is provided.

Methodology

Sample preparation	mESCs plated on inactivated MEFs were treated with 4-hydroxytamoxifen (4-OHT) for 2 days and after 1.5 days of growth in SRES without 4-OHT, H2B-EGFP+ mESCs, resulting from a successful CreERT2-mediated recombination that occurred during 4-OHT treatment, were sorted by FACS (Fluorescence-Activated Cell Sorting).
Instrument	The cell sorting was done using BD FACS Aria; flow cytometry analysis was done using BD LSR II and Fortessa.
Software	The data were collected using FACS Diva software and analyzed using FlowJo Software (Version 9.7.6 and version 10.3.0)
Cell population abundance	The samples used for RNA-seq library construction were sorted and the purity is >99% determined by post-sort flow cytometry analysis; the purity of samples purified using dynabeads is >98-98.5% determined by flow cytometry analysis.
Gating strategy	Live/Dead dye and Thy1.2 negative, CD326+EGFP+ population representing the mESCs populations responsive to the tamoxifen treatment.

☒ Tick this box to confirm that a figure exemplifying the gating strategy is provided in the Supplementary Information.
